# His Fiftieth Anniversary

**Published:** 1898-08

**Authors:** 


### His Fiftieth Anniversary.—The Venerable Dr. S. H. Stout Presented With a Gold-Headed Cane.

Last night, July 17 '98, the fiftieth anniversary since the graduation in medicine of Dr. S. H. Stout, the members of the Dallas Medical and Surgical Society called at his residence and through its committee, composed of Drs. E. Aronson, S. Eagon, S. W. McJunkin, J. B. Smoot and J. W. Lane, presented the gentleman with a handsome gold-headed cane. The presentation speech by Dr. Eagon was as follows:

"Most worthy and venerable brother: In behalf of the Dallas Medical and Surgical association and as a member of a committee appointed by this society, of which you are yourself an honorary member, it becomes my valued privilege on this happy occasion of your golden jubilee—fifty years of successful and distinguished labor and devotion to science and humanity—to present to you in the name of our society the beautiful instrument which I hold in my hand as an expression of the high esteem, admiration and fraternal affection in which you are held by the medical profession of Dallas. Fifty years of medical and surgical practice carries us back to the preanaesthetic period, and takes within its scope antisepsis and brings us up face to face with the marvelous developments of bacteriology, on which is based sanitary science or public hygiene, marking the three most important epochs in the history of medicine.

"Endowed with extraordinary native capacities, both intellectual and moral, refined by the highest order of literary and scientific training, your equipment for a distinguished medical career was complete at the beginning. Fifty years of professional observation and clinical experience, both in civil and in military practice, have afforded you vast opportunities for gathering sheaves for the garner of medical science, such as fall to the lot of but few, indeed. And now well advanced in your professional career it must afford you happiness beyond expression to reflect that these grand opportunities have been turned to best account for science and humanity. Thrice happy is he who accomplishes well his life work. But the labor, the care, the anxiety, the fears, the keenly-felt responsibilities, the self-sacrificing devotion to sacred duty that such a noble life involves can be correctly measured out by one who, in some measure, has shared these treats, which after all only serve to ennoble, refine and elevate us to the supreme dignity of the true physician. This society regards you as a venerable Nestor of our grand profession, as a Gamaliel at whose feet we feel proud to sit; as one who has ever regarded our profession, not as a trade, but as a great morality; not as a business, but as a mission appointed by God for the benefit of the children of men. Nothing brings a man so near the gods as giving health to his fellow-man. Then, most worthy and venerable brother, take this token of esteem and affection and cherish it. Let it ever remind you of the friends who presented it, and let it ever be a monitor to you to continue a worthy minister at the altar of your choice.

Dr. Stout responded as follows:

"It is indeed a surprise to meet so many of the medical fraternity here tonight, who have spontaneously come here to honor me by their congratulations upon this anniversary of my graduation as a doctor of medicine. That you have taken me unawares is all the more pleasing and gratifying. Of the many honors conferred upon me by the unsought action of my professional brethren this occasion and the purpose which brought you here tonight touches my feelings most profoundly. To have received from such a body of professional men the verdict of 'well done' after a career of fifty years of practice and association with noble, generous men of the noblest, most unselfish and most charitable profession known to civilized life is indeed a compliment of which any man may be justly proud. That I have not been faultless is to claim no more than can be justly claimed for any man in this life. To say that I havə erred in my efforts to hold high the banner of professional honor, not only in my intercourse with my clientelle and general public, but also with my professional brethren is to acknowledge that I am human. Never, however, have I failed or faltered in advocating the grand, holy mission of our profession or envied any professional brother's success and honors. Fifty-nine years ago I entered with all the zeal of young manhood upon the study of medicine and at this day my zeal in the pursuit of it has not abated. No one of you who are my juniors can realize as I do the wonderful progress made in the science of medicine in all its branches and the clinical application of its principles. Every step of that progress it has been an every day's delight to recognize, and as the shadows of my life's evening are rapidly lengthening the chief regret I feel, when I take account of my expectation of life, is that I cannot hope to witness the developments which the experiences of the last half century assure us will be made in the next fifty years. But I desist from the temptation to rehearse anew a moiety of the many discoveries made in the science and improvements in the application of its principles in the period of my professional career. I have alluded to them to emphasize my estimation of the nobility and honorable record of our profession and the noble work done by those who in my time have studied and practiced it. You, gentlemen, are a part of that great army of men, who throughout the civilized world are toiling and studying to save and prolong human life and to ameliorate human suffering, and I know you are representatives of the best material of that great army. Therefore, the compliment you pay me tonight I feel to be the highest honor ever conferred upon me, and your presence here gives assurance that our friendship is reciprocal and our mutual sympathies sincere for each and every one of you. Ever since I began my sojourn in Dallas you have ever as now commanded my respect, but tonight you are entitled to and have my sincere affection, which it shall be no fault of mine if it does not grow day by day in intensity until the sod of the valley shall have hidden my form from view. May that same Providence that has so kindly vouchsafed to me and mine so many privileges and blessings watch over you and yours."

